# Duclauxin Derivatives From Fungi and Their Biological Activities

**DOI:** 10.3389/fmicb.2021.766440

**Published:** 2021-12-22

**Authors:** Hamza Shahid, Teng Cai, Yuyang Wang, Caiqing Zheng, Yuting Yang, Ziling Mao, Ping Ding, Tijiang Shan

**Affiliations:** ^1^Guangdong Key Laboratory for Innovative Development and Utilization of Forest Plant Germplasm, College of Forestry and Landscape Architecture, South China Agricultural University, Guangzhou, China; ^2^School of Pharmaceutical Sciences, Guangzhou University of Chinese Medicine, Guangzhou, China

**Keywords:** duclauxin derivatives, fungi, secondary metabolites, biological activities, biosynthesis

## Abstract

Duclauxin is a heptacyclic oligophenalenone dimer consisting of an isocoumarin and a dihydroisocoumarin unit. These two tricyclic moieties are joined by a cyclopentane ring to form a unique hinge or castanets-like structure. Duclauxin is effective against numerous tumor cell lines because it prevents adenosine triphosphate (ATP) synthesis by inhibiting mitochondrial respiration. There are about 36 reported natural duclauxin analogs mainly produced by 9 *Penicillium* and *Talaromyces* species (*T. duclauxii*, *T. aculeatus*, *T. stipitatus*, *T. bacillisporus*, *T. verruculosus*, *T. macrosporus*, *P. herquei*, *P. manginii*, and *Talaromyces* sp.). These metabolites exhibit remarkable biological activities, including antitumor, enzyme inhibition, and antimicrobial, showing tremendous potential in agricultural and medical applications. This review highlights the chemical structures and biological activities of fungal duclauxins, together with biosynthesis, absolute configuration, and mode of action for important duclauxins. Furthermore, phylogenetic analysis and correct names of *Penicillium* and *Talaromyces* species producing duclauxins are presented in this review.

## Introduction

Natural products are derived from natural sources such as microorganisms, plants, or animals (Jeerapong et al., 2015; Calixto, 2019; Mao et al., 2021). In the past few years, it has been proved that they are a continuing source of novel bioactive compounds and have a significant impact on modern drug discovery (Singh and Chandra, 2014; Abbas et al., 2018). At present, more than 70% of anticancer and antibacterial compounds are natural products or their derivatives that play a vital role in the pharmaceutical sector (Newman and Cragg, 2016, 2020). Among the microorganisms, fungi have attained much attention in recent decades, mainly regarding the discovery of beneficial secondary metabolites. It is estimated that more than 23,000 microbial natural products have been discovered, of which around 45% are from fungi (Cao et al., 2015). Fungal secondary metabolites have wide-ranging applications such as antifungals, antibiotics, antiparasitic, and anticancer agents (Barbero et al., 2018; Lima et al., 2019; Shan et al., 2020; Torres-Mendoza et al., 2020). The most famous example in history is the discovery of penicillin, an antibiotic drug produced by *Penicillium chrysogenum* that has saved many human lives (Fleming, 1929). Genera *Penicillium* and *Talaromyces* produce a wide range of polyketide-derived secondary metabolites with diverse bioactivities. These compounds are promising antitumor and antibacterial agents, leading to their applications in agriculture, environment, forestry, and pharmaceutical sectors (Cao et al., 2015; Chaudhary et al., 2020; Dramae et al., 2020; Jimenez-Arreolaa et al., 2020).

Among fungal secondary metabolites, duclauxin (**1**) is a well-known antitumor agent because it prevents ATP synthesis by inhibiting mitochondrial respiration (Fuska et al., 1974). Structurally, it is a heptacyclic oligophenalenone dimer consisting of an isocoumarin and a dihydroisocoumarin core (Shibata et al., 1965; Ogihara et al., 1968). Duclauxin (**1**) was first isolated from the cultures of fungus *Talaromyces duclauxii* in 1965 (Shibata et al., 1965). After that, a growing number of duclauxin derivatives were isolated from the genera *Talaromyces* and *Penicillium* (Yamazaki and Okuyama, 1980; Wu et al., 2015; Zang et al., 2016a; Chaiyosang et al., 2019; Wang et al., 2019; Chaudhary et al., 2020; Dramae et al., 2020; Jimenez-Arreolaa et al., 2020). Duclauxins have attracted particular attention as they have great potential to become remedies against multiple diseases. These compounds possess a wide range of biological properties, including antitumor (Cao et al., 2015), antibacterial (Dramae et al., 2020), cytotoxic (Dethoup et al., 2006), antimalarial (Chaiyosang et al., 2019), and enzyme inhibition (Wu et al., 2015; Wang et al., 2019; Jimenez-Arreolaa et al., 2020). Some of these compounds have been identified as novel inhibitors of cancer cell lines (Shiojiri et al., 1983) and pathogens (Chaiyosang et al., 2019). Thus, they can potentially contribute to the development of new cancer chemotherapies and antibiotics.

This review focuses on chemical structures and biological activities of duclauxin derivatives produced by fungi. Meanwhile, the phylogenetic relationship of *Penicillium* and *Talaromyces* species producing duclauxins is described as well. Furthermore, biosynthesis, absolute configuration, and mode of action for some duclauxin derivatives are also discussed here. This review covers the literature related to duclauxins from the year of 1965–2021.

## Duclauxin and Its Derivatives Produced by Fungi

**FIGURE 1:**
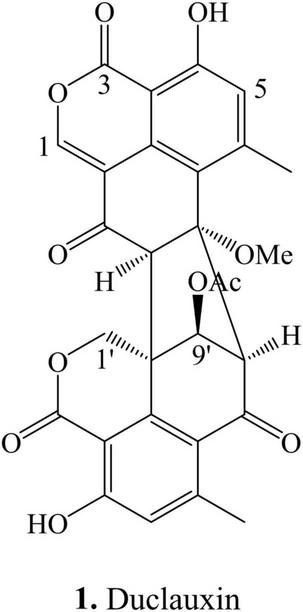
Structural representation of duclauxin (**1**).

Duclauxin (**1**) (Figure 1) is a heptacyclic bis (oxaphenalenone) heterodimer made up of two functionalized 2-oxaphenalen-1-one units (corymbiferan skeleton) linked by two bonds, which form the cyclopentane. Interestingly, all duclauxin derivatives contain at least one unit of the dihydrocoumarin benzo[de]isochromen-1(3*H*)-one, and most of them are characterized by a heptacyclic ring system. As shown in Table 1, duclauxin (**1**) was further produced by seven strains from different *Peniclillium* and *Talaromyces* species since the first duclauxin (**1**) isolated from *T. duclauxii* in 1965. Until now, 36 duclauxin derivatives are mainly produced by *Talaromyces* and *Penicillium* species, isolated from marine sponges, plants endophytes, soil samples, mangroves, and coral reefs (Table 1).

**TABLE 1:** Isolation source of duclauxin derivatives.

Compound	Fungus	Isolation source	Localization	Medium	References
Duclauxin (**1**)	*T. duclauxii* (previous name *P. duclauxii)*	–	–	Czapek-Dox medium	Shibata et al., 1965
	*T. bacillisporus*	Soil	Thailand	Rice medium	Dethoup et al., 2006
	*T. stipitatus* (previous name *P*. *stipitatum*)	–	–	–	Kuhr et al., 1973
	*P. herquei* ATCC 34665	–	–	Gostar medium	Bryant et al., 1993
	*T. stipitatus*	Soil	Australia	Pearl barley	Chaudhary et al., 2020
	*P. manginii* YIM PH30375	Elder root of *Panax notoginseng*	China	–	Cao et al., 2015
	*Talaromyces* sp. IQ-313	Anthill soil	Mexico	Cheerios medium	Jimenez-Arreolaa et al., 2020
	*T. verruculosus*	Soft coral marine derived fungus	China	Liquid medium	Wang et al., 2019
Bacillisporin A (**2**)	*T. bacillisporus*	–	–	Malt extract medium	Yamazaki and Okuyama, 1980
		Soil	Thailand	Rice medium	Dethoup et al., 2006
		Soil	Thailand	PDB medium	Dramae et al., 2020
	*T. macrosporus* KKU-1NK8	Forest soil	Thailand	Malt extract peptone broth	Chaiyosang et al., 2019
	*T. stipitatus* ATCC 10500	Soil	–	Polished rice	Zang et al., 2016b
	*T. aculeatus* 9EB (previous name *P. aculeatum*)	Leaves of mangrove *K. candel*	China	Rice medium	Huang et al., 2017
Bacillisporin B (**3**)	*T. bacillisporus*	–	–	Malt extract medium	Yamazaki and Okuyama, 1980
		Soil	Thailand	Rice medium	Dethoup et al., 2006
		Soil	Thailand	PDB medium	Dramae et al., 2020
	*T. macrosporus* KKU-1NK8	Forest soil	Thailand	Malt extract peptone broth	Chaiyosang et al., 2019
	*T. aculeatus* 9EB (previous name *P. aculeatum*)	Leaves of mangrove *K. candel*	China	Rice medium	Huang et al., 2017
Bacillisporin I (**4**)	*T. bacillisporus* BCC17645	Soil fungus	Thailand	PDB medium	Dramae et al., 2020
Bacillisporin J (**5**)	*T. bacillisporus* BCC17645	Soil fungus	Thailand	PDB medium	Dramae et al., 2020
Bacillisporin D (**6**)	*T. bacillisporus*	Soil	Thailand	Rice medium	Dethoup et al., 2006
	*T. macrosporus* KKU-1NK8	Forest soil	Thailand	Malt extract peptone broth	Chaiyosang et al., 2019
Bacillisporin E (**7**)	*T. bacillisporus*	Soil	Thailand	Rice medium	Dethoup et al., 2006
	*T. macrosporus* KKU-1NK8	Forest soil	Thailand	Malt extract peptone broth	Chaiyosang et al., 2019
9a-*epi*-Bacillisporin E (**8**)	*T. stipitatus* ATCC 10500	Soil	–	Polished rice	Zang et al., 2016b
Bacillisporin F (**9**)	*T. verruculosus*	Soft coral *Goniopora* sp.	China	Liquid medium	Wang et al., 2019
	*T. stipitatus* ATCC 10500	Soil fungus	–	Polished rice	Zang et al., 2016b
	*T. macrosporus* KKU-1NK8	Forest soil	Thailand	Malt extract peptone broth	Chaiyosang et al., 2019
1-*epi*-Bacillisporin F (**10**)	*T. stipitatus* ATCC 10500	Soil	–	Polished rice	Zang et al., 2016b
	*T. macrosporus* KKU-1NK8	Forest soil	Thailand	Malt extract peptone broth	Chaiyosang et al., 2019
Macrosporusone E (**11**)	*T*. *macrosporus* KKU-1NK8	Forest soil	Thailand	Malt extract peptone broth	Chaiyosang et al., 2019
1-*epi*-macrosporusone E (**12**)	*T*. *macrosporus* KKU-1NK8	Forest soil	Thailand	Malt extract peptone broth	Chaiyosang et al., 2019
Cryptoclauxin (**13**)	*T. duclauxii* (previous name *P. duclauxii*)	–	–	–	Ogihara et al., 1966
	*T. stipitatus*	–	–	MEPA medium	Gao et al., 2018
Macrosporusone D (**14**)	*T*. *macrosporus* KKU-1NK8	Forest soil	Thailand	Malt extract peptone broth	Chaiyosang et al., 2019
Xenoclauxin (**15**)	*T. duclauxii* (previous name *P. duclauxii*)	–	–	–	Ogihara et al., 1966
	*Talaromyces* sp. IQ-313	Anthill soil	Mexico	Cheerios medium	Jimenez-Arreolaa et al., 2020
	*T. macrosporus* KKU-1NK8	Forest soil	Thailand	Malt extract peptone broth	Chaiyosang et al., 2019
	*T. verruculosus*	Soft coral *Goniopora* sp.	China	Liquid medium	Wang et al., 2019
Talaromycesone A (**16**)	*Talaromyces* sp. LF458	Sponge of *A. verrucosa*	Italy	WSP30TM medium	Wu et al., 2015
Verrucolosin B (**17**)	*T. verruculosus*	Soft coral *Goniopora* sp.	China	Liquid medium	Wang et al., 2019
Talaroketal A (**18**)	*T. stipitatus* ATCC 10500	Soil	–	Rice soy-pepton	Zang et al., 2016a
Talaroketal B (**19**)	*T. stipitatus* ATCC 10500	Soil	–	Rice soy-pepton	Zang et al., 2016a
Verrucolosin A (**20**)	*T. verruculosus*	Soft coral *Goniopora* sp.	China	Liquid medium	Wang et al., 2019
Bacillisporin H (**21**)	*T. stipitatus* ATCC 10500	Soil	–	Polished rice	Zang et al., 2016b
Duclauxamide A1 (**22**)	*P. manginii* YIM PH30375	Elder root of *P. notoginseng*	China	–	Cao et al., 2015
	*T. bacillisporus* BCC17645	Soil	Thailand	PDB medium	Dramae et al., 2020
Duclauxamide B (**23**)	*T. bacillisporus* BCC17645	Soil fungus	Thailand	PDB medium	Dramae et al., 2020
Duclauxamide C (**24**)	*T. bacillisporus* BCC17645	Soil fungus	Thailand	PDB medium	Dramae et al., 2020
Talauxin E (**25**)	*T. stipitatus*	Soil	Australia	Pearl barley	Chaudhary et al., 2020
Talauxin Q (**26**)	*T. stipitatus*	Soil	Australia	Pearl barley	Chaudhary et al., 2020
Talauxin V (**27**)	*T. stipitatus*	Soil	Australia	Pearl barley	Chaudhary et al., 2020
Talauxin L (**28**)	*T. stipitatus*	Soil	Australia	Pearl barley	Chaudhary et al., 2020
Talauxin I (**29**)	*T. stipitatus*	Soil	Australia	Pearl barley	Chaudhary et al., 2020
Bacillisporin C (**30**)	*T. bacillisporus*	–	–	Malt extract medium	Yamazaki and Okuyama, 1980
		Soil	Thailand	Rice medium	Dethoup et al., 2006
		Soil	Thailand	PDB medium	Dramae et al., 2020
Bacillisporin G (**31**)	*T. stipitatus* ATCC 10500	Soil	–	Polished rice	Zang et al., 2016b
	*Talaromyces* sp. IQ-313	Anthill soil	Mexico	Cheerios medium	Jimenez-Arreolaa et al., 2020
	*T*. *macrosporus* KKU-1NK8	Forest soil	Thailand	Malt extract peptone broth	Chaiyosang et al., 2019
Macrosporusone A (**32**)	*T*. *macrosporus* KKU-1NK8	Forest soil	Thailand	Malt extract peptone broth	Chaiyosang et al., 2019
Macrosporusone B (**33**)	*T*. *macrosporus* KKU-1NK8	Forest soil	Thailand	Malt extract peptone broth	Chaiyosang et al., 2019
Macrosporusone C (**34**)	*T*. *macrosporus* KKU-1NK8	Forest soil	Thailand	Malt extract peptone broth	Chaiyosang et al., 2019
Talaromycesone B (**35**)	*Talaromyces* sp. LF458	Sponge of *A. verrucosa*	Italy	WSP30TM medium	Wu et al., 2015
	*Talaromyces* sp. IQ-313	Anthill soil	Mexico	Cheerios medium	Jimenez-Arreolaa et al., 2020
	*T. macrosporus* KKU-1NK8	Forest soil	Thailand	Malt extract peptone broth	Chaiyosang et al., 2019
Talaromycesone C (**36**)	*T*. *macrosporus* KKU-1NK8	Forest soil	Thailand	Malt extract peptone broth	Chaiyosang et al., 2019

*“–” means nil.*

**FIGURE 2:**
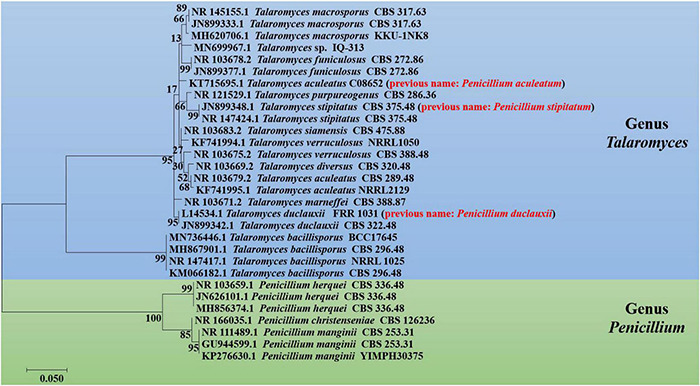
Phylogenetic tree of *Penicillium* and *Talaromyces* species regarding duclauxins, based on the rDNA-ITS sequence obtained from the NCBI database. The tree was constructed with the maximum likelihood method in MEGA 7.0 using default parameters, and bootstrap values were calculated after 1,000 replications. The previous or invalid species name is presented with red color between the parenthesis.

*Penicillium* and *Talaromyces* are economically, biotechnologically, and medically important genera that belong to the Eurotiales order and contain many species possessing a worldwide distribution and a vast range of ecological habitats (Tsang et al., 2018). Many *Penicillium* and *Talaromyces* species are vital to the pharmaceutical industries as they are robust producers of a diverse spectrum of secondary metabolites with vigorous biological activities (Cai et al., 2020; Rahman and Sarker, 2020). Over the past few years, the taxonomic history of anamorphic species attributed to *Penicillium* subgenus *Biverticillium* was reviewed and transferred to the genus *Talaromyces* (Houbraken et al., 2020). Since taxonomy is a dynamic discipline, and name changes of fungi with medical, biotechnological, and industrial importance are often challenging for researchers in the applied fields to understand. As a result, we added the list of accepted species present in genera *Penicillium* and *Talaromyces* that produced duclauxins, based on ITS, *BenA*, *CaM* and *RPB2* multigene data obtained from the NCBI nucleotide database (GenBank). The list includes accepted names, basionyms, subgenus, section, series, and reproduction attributed to genera *Penicillium* and *Talaromyces* (Table 2). To confirm the previous findings, the phylogenetic relationship of *Penicillium* and *Talaromyces* species producing duclauxins was analyzed based on the rDNA-ITS sequence obtained from the NCBI database (GenBank). Figure 2 shows the relationship among the species belonging to genera *Penicillium* and *Talaromyces*. The phylogenetic tree (Figure 2) revealed that the strain *T. duclauxii* (accession no. L14534.1, previous name: *P. duclauxii*) formed a clade with the strain *T. duclauxii* (accession no. JN899342.1) and had 98.07% similarity. Similarly, the strain *T. stipitatus* (accession no. JN899348.1), previously known as *P. stipitatum* had 100% similarity with the strain *T. stipitatus* (accession no. NR147424.1) and clustered on the same branch. Moreover, the strain *T. aculeatus* C08652 (accession no. KT715695.1) was first identified as *P. aculeatum* based on the 18S rDNA sequence analysis (Huang et al., 2017). However, the latest literature implies that the name of *P. aculeatum* has been changed into *T. aculeatus* (Houbraken et al., 2020). The phylogenetic tree (Figure 2) also revealed that the strain *T. aculeatus* C08652 (accession no. KT715695.1) formed a clade with members of the genus *Talaromyces*. These results confirmed that species attributed to *Penicillium* subgenus *Biverticillium* (*P. aculeatum*, *P. stipitatum*, and *P. duclauxii*) are phylogenetically related to the genus *Talaromyces* (Figure 2) and were consistent with the article recently published by Houbraken and coworkers (Houbraken et al., 2020). Therefore, we used the currently accepted names of *Penicillium* and *Talaromyces* species in this review that produced duclauxins. Based on the structural skeletons, duclauxin derivates can be classified into four types including heptacyclic, octal- and nona-cyclic, *N*-containing, and asymmetrical.

**TABLE 2:** Taxonomy and nomenclature of *Penicillium* and *Talaromyces* species.

Current accepted name	Basionym	Subgenus	Section	Series	Reproduction	GenBank accession number	References
*P. herquei*	–	*Aspergilloides*	*Sclerotiorum*	*Herqueorum*	Asexual	JN626101(**ITS**) JN625970(***BenA***) JN626013(***CaM***) JN121494(***RPB2***)	Houbraken et al., 2020
*P. manginii*	–	*Aspergilloides*	*Citrina*	*Westlingiorum*	Asexual	GU944599(**ITS**) JN606651(***BenA***) MN969274(***CaM***) JN606618(***RPB2***)	Houbraken et al., 2020
*T. aculeatus*	*P. aculeatum*	–	*Talaromyces*	–	Asexual	KF741995(**ITS**) KF741929(***BenA***) KF741975(***CaM***) MH793099(***RPB2***)	Houbraken et al., 2020
*T. bacillisporus*	*P. bacillisporum*	–	*Bacillispori*	–	Homothallic	KM066182(**ITS**) AY753368(***BenA***) KJ885262(***CaM***) JF417425(***RPB2***)	Houbraken et al., 2020
*T. duclauxii*	*P. duclauxii*	–	*Talaromyces*	–	Asexual	JN899342(**ITS**) JX091384(***BenA***) KF741955(***CaM***) JN121491(***RPB2***)	Houbraken et al., 2020
*T. macrosporus*	*T. flavus* var. *macrosporus*	–	*Talaromyces*	–	Homothallic	JN899333(**ITS**) JX091382(***BenA***) KF741952(***CaM***) KM023292(***RPB2***)	Houbraken et al., 2020
*T. stipitatus*	*P. stipitatum*	–	*Talaromyces*	–	Homothallic	JN899348(**ITS**) KM111288(***BenA***) KF741957(***CaM***) KM023280(***RPB2***)	Houbraken et al., 2020
*T. verruculosus*	*P. verruculosum*	–	*Talaromyces*	–	Asexual	KF741994(**ITS**) KF741928(***BenA***) KF741944(***CaM***) KM023306(***RPB2***)	Houbraken et al., 2020

*“–” means nil.*

***ITS** stands for “internal transcribed spacer regions”.*

***BenA** stands for “partial beta-tubulin gene region”.*

***CaM** stands for “calmodulin gene region”.*

***RPB2** stands for “RNA polymerase II second largest subunit”.*

### Heptacyclic Duclauxin Derivatives

**FIGURE 3:**
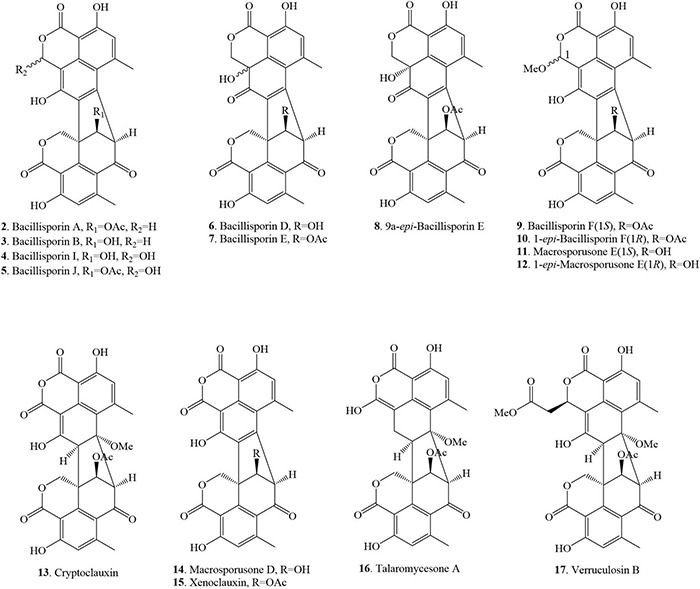
Structural representation of heptacyclic duclauxin derivatives.

The structures of heptacyclic duclauxins are described as two tricyclic moieties joined by a cyclopentane ring to form a unique hinge-like structure. About 15 heptacyclic duclauxin derivatives were isolated from several fungal strains dwelling in different ecological niches, such as soil, mangrove endophyte, and marine environment (Figure 3 and Table 1). Two new duclauxin derivatives bacillisporins A-B (**2–3**), were isolated from the ether (Et_2_O) extract of mycelia of *T. bacillisporus* NHL 2660 grown on a malt extract medium (Yamazaki and Okuyama, 1980). Afterwards, bacillisporin A (**2**) was re-isolated from *T. stipitatus* grown on polished rice medium (Zang et al., 2016b). In another report, bacillisporins A (**2**) and B (**3**) were produced by fungus *T. macrosporus* KKU-1NK8 (Chaiyosang et al., 2019). Interestingly, bacillisporins A (**2**) and B (**3**) were isolated from a mangrove-derived endophytic fungus *T. aculeatus* No. 9EB for the first time. The fungus was obtained from the leaves of mangrove *Kandelia candel*, collected from Yangjiang, Guangdong province, China (Huang et al., 2017). Further investigations revealed compounds **2** and **3** have also been discovered from the soil fungus *T. bacillisporus*. The continuing research on duclauxin-like secondary metabolites from *T. bacillisporus* grown on potato dextrose broth (PDB) media led to the discovery of two new duclauxin derivatives, namely bacillisporins I (**4**) and J (**5**), respectively. The skeleton of compounds **2**, **3**, **4**, and **5** is identical except for minor differences at C-9′ and C-1 positions. However, the absolute configuration of compounds **4** and **5** at C-1 could not be determined due to the similar CD patterns of both 1*S* and 1*R* configurations. Thus the ^1^H chemical shift calculation together with DP4 probability analysis may be required to confirm the absolute configuration at C-1 (Figure 3). Heptacyclic oxyphenalenone dimers, bacillisporins D (**6**) and E (**7**) were reported first from a soil fungus *T. bacillisporus* (Dethoup et al., 2006; Dramae et al., 2020). With time, compounds **6–7** were acquired by the chemical investigation of *T. macrosporus* KKU-1NK8 grown on malt extract peptone broth. The only difference between the structures of compounds **6** and **7** was the C-9′ position (“OH” group for compound **6** and “OAc” group for compound **7**). However, the absolute configuration of compounds **6** and **7** at C-9a has not been determined until now. Further experiments such as ^1^H chemical shift calculation and DP4 probability analysis may be required to confirm the absolute configuration at C-9a (Chaiyosang et al., 2019).

Three new polyketide-derived oligophenalenone dimers, 9a-*epi*-bacillisporin E (**8**), bacillisporin F (**9**), and 1-*epi*-bacillisporin F (**10**) were isolated from the soil fungus *T. stipitatus* ATCC 10500. Their absolute configurations were determined using Gauge-Independent Atomic Orbital (GIAO) Nuclear Magnetic Resonance (NMR) shift calculation followed by DP4 analysis. The differences between compounds **9** and **10** were the chemical shifts of the methoxy group in CD_3_OD (δ_*H*_ 3.66 for **9** vs. δ_*H*_ 3.58 for **10**) and the methine H-1 (δ_*H*_ 6.57 for **9** vs. δ_*H*_ 6.55 for **10**) (Zang et al., 2016b). Subsequently, bacillisporin F (**9**) and 1-*epi*-bacillisporin F (**10**) have also been found in soil fungus *T. macrosporus.* In addition, compound **9** was isolated from a marine fungus *T. verruculosus* in soft coral. The resonance signals of bacillisporin F (**9**) and *1-epi*-bacillisporin F (**10**) were similar to those of the macrosporusone E (**11**) and 1-*epi*-macrosporusone E (**12**), except for a hydroxyl group at C-9′ being replaced by an acetoxyl group (Chaiyosang et al., 2019; Wang et al., 2019; Table 1). Cryptoclauxin (**13**), a well-known duclauxin derivative, was isolated from *T. duclauxii* for the first time using the silicic acid column chromatography technique (Ogihara et al., 1966). This compound was also re-isolated from *T. stipitatus* in 2018 (Gao et al., 2018).

Forest soil fungus *T. macrosporus* KKU-1NK8 yielded three new polyketide-derived oxaphenalenone dimers named macrosporusone E (**11**), 1-*epi*-macrosporusone E (**12**), and macrosporusone D (**14**) along with xenoclauxin (**15**). Their structures were established by spectroscopic data and electronic circular dichroism (ECD) analyses. The structure of compound **14** was very similar to that of compound **15**, except that an acetoxyl group at C-9′ in **15** was replaced by a hydroxyl group in **14**. The sole difference between compounds **11** and **12** was the configuration of C-1 (1*S* for **11** and 1*R* for **12**). However, the numerical misrepresentation of macrosporusone E (**12**) and macrosporusone D (**14**) was found in the published article (10.1016/j.fitote.2019.03.015) that caused considerable confusion to the readers (Chaiyosang et al., 2019). Further studies also resulted in the identification of xenoclauxin (**15**) from *T. verruculosus*, *T. duclauxii* and a soil fungus *Talaromyces* sp. IQ-313 (Ogihara et al., 1966; Wang et al., 2019; Jimenez-Arreolaa et al., 2020).

Chemical investigation of marine fungus *Talaromyces* sp. LF458, isolated from a sponge *Axinella verrucosa* collected from the Mediterranean Sea, Italy, afforded talaromycesone A (**16**) (Wu et al., 2015). The continuous research on marine fungus resulted in the discovery of verrucolosin B (**17**) from a soft coral-associated fungus *T. verruculosus* collected from the South China Sea. The structure of compound **17** presents a unique methoxycarbonyl-methyl moiety at the C-1 position (Wang et al., 2019; Figure 3).

### Octal- and Nona-Cyclic Duclauxin Derivatives

**FIGURE 4:**
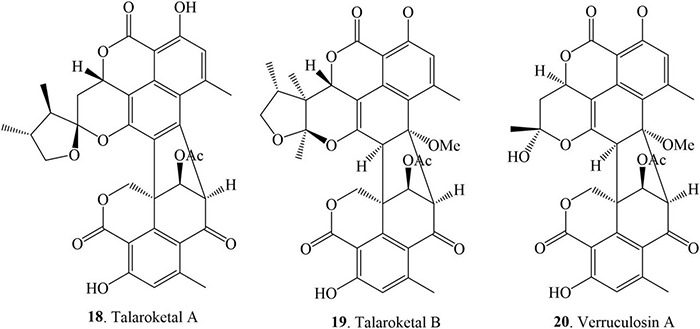
Structural representation of octal- and nona-cyclic duclauxin derivatives.

This class of compounds comprises tetra- and penta-cyclic moieties on one side of the structure joined *via* two C-C bonds with the tricyclic benzannulated moieties. Only three compounds have been reported in this class and they were isolated from several sources of genus *Talaromyces* (Figure 4 and Table 1). Talaroketals A (**18**) and B (**19**) were isolated from the soil fungus *T. stipitatus* ATCC 10500. Their structures and absolute configurations were determined based on X-ray diffraction and ECD experiments. Talaroketals A (**18**) and B (**19)** represent the first example of bis (oxaphenalenone) 5,6-spiroketal and 5,6-fused ketal, respectively. Compound **18** is a new member of the rare benzannulated [6,5]-spiroketal class of natural products (Zang et al., 2016a). Chemical investigation of the marine-derived fungus, *T. verruculosus*, isolated from the soft coral *Goniopora* sp. collected from the South China Sea, yielded a new oligophenalenone dimer, named verrucolosin A (**20**). The detailed structure and absolute configuration were solved with the aid of spectroscopic data, X-ray crystallography, optical rotation, ECD analysis, and NMR calculations. Compound **20** possesses a unique/distinctive octacyclic skeleton in the oxaphenelenone dimer family (Wang et al., 2019).

### Nitrogen-Containing Duclauxin Derivatives

**FIGURE 5:**
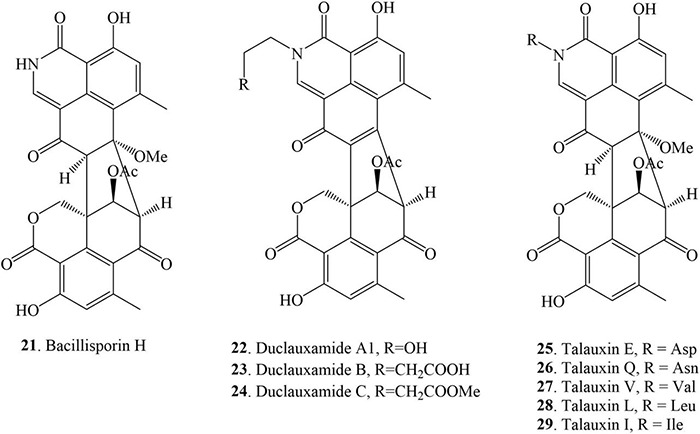
Structural representation of *N*-containing duclauxin derivatives.

Nitrogen-containing duclauxin derivatives are scarcely found in nature, and thus until now, 9 *N*-containing duclauxin derivatives have been discovered from nature, as shown in Table 1 and Figure 5. The structures of these compounds consist of a heptacyclic dimer in which a nitrogen atom is present in the upper 6/6/6 tri-ring system. Bacillisporin H (**21**), a new nitrogenated bis-oxaphenalenone was isolated from EtOAc extract of *T. stipitatus* obtained from rice-based media (Zang et al., 2016b). Studies carried out by Cao et al. (2015) presented the isolation of new polyketide-derived heptacyclic oligophenalenone dimer duclauxamide A1 (**22**) bearing an *N*-2-hydroxyethyl moiety from *P. manginii* YIM PH30375, which was isolated from the elder root of *Panax notoginseng*, China. X-ray single-crystal diffraction technique and ^13^C NMR DFT calculation confirmed that compound **22** and other duclauxin analogs possess a unified *S*-configuration at C-9′, which corrected a long-standing misrepresentation of duclauxin and its analogs as C-9′ *R* epimers (Cao et al., 2015). A few years later, duclauxamide A1 (**22**) and two new *N*-containing oxaphenalenone dimers, duclauxamides B-C (**23–24**), were discovered from the soil fungus *T. bacillisporus* BCC17645 (Dramae et al., 2020). Five new oxyphenalenone amino acid hybrids, talauxins E, Q, V, L, and I (**25–29**), have been identified from the culture of *T. stipitatus* FP2248C on pearl barley medium. The fungus was recovered as a quiescent contaminant from the Australian fungus *Aspergillus banksianus* isolated from a soil sample collected in Collaroy, New South Wales, Australia (Chaudhary et al., 2020).

### Asymmetrical Duclauxin Derivatives

**FIGURE 6:**
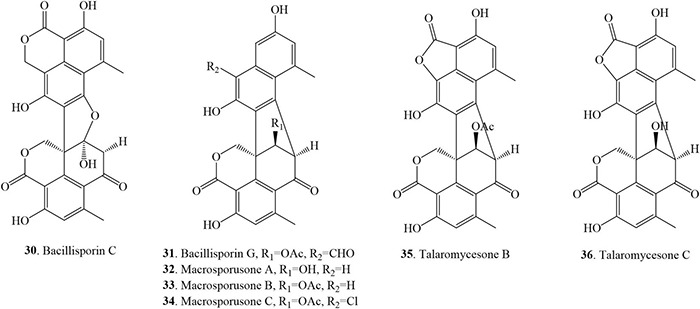
Structural representation of asymmetrical duclauxin derivatives.

This class of monomer compounds presents an irregular shape in their structures as compared to other duclauxin derivatives. 7 duclauxins have been identified in this class from fungi so far (Figure 6 and Table 1). Bacillisporin C (**30**) was isolated for the first time from the mycelia of *T. bacillisporus* NHL 2660 grown on malt extract medium (Yamazaki and Okuyama, 1980). Likewise, isolation of bacillisporin C (**30**) was reported twice from soil fungus *T. bacillisporus.* Compound **30** was declared as the first compound in the duclauxin family, in which one C-C bond is connected with oxygen (O) between the two tricyclic moieties of a heptacyclic dimer (Dethoup et al., 2006; Dramae et al., 2020). Chemical evaluation of a soil fungus *T. stipitatus* ATCC 10500 collected from Thailand afforded bacillisporin G (**31**) (Zang et al., 2016b). With time, compound **31** was reported from other *Talaromyces* species such as *Talaromyces* sp. IQ-313 and *T. macrosporus* KKU-1NK8, both were isolated from soil (Chaiyosang et al., 2019; Jimenez-Arreolaa et al., 2020). Chromatographic separation of the EtOAc extract of a forest soil fungus *T. macrosporus* KKU-1NK8 resulted in three new polyketide-derived oxaphenalenone dimers, macrosporusones A-C (**32–34**). The fungus was isolated from the forest soil collected in the Pha Nok Kao Silvicultural Station, Khon Kaen Province, Thailand. The NMR spectroscopic data of compound **32** were similar to bacillisporin G (**31**) except for the absence of an aldehyde group at C-9a and that a hydroxyl group replaced an acetyl group at C-9′. The ^1^H and ^13^C NMR spectroscopic data of compound **33** were similar to those of compound **32**, except that the hydroxyl group at C-9′ was replaced by an acetoxyl group (δ_*C*_ carbonyl ester group 170.6 and methyl group δ_*H/C*_ 1.99 (s)/20.8). Macrosporusone C (**34**) is the first oxaphenalenone dimer reported with a chlorine atom in the molecule (Chaiyosang et al., 2019).

Talaromycesone B (**35**) was isolated as a brown powder from the culture broth and mycelia of a marine fungus *Talaromyces* sp. LF458 on WSP30 medium. Strain LF458 was isolated from tissues of the sponge *Axinella verrucosa* collected from the Mediterranean Sea, Italy, and tentatively classified as a *T. funiculosum*. Compound **35** represents the first case of a 1-nor oxaphenalenone dimer carbon skeleton in nature (Wu et al., 2015; Imhoff, 2016). Additionally, talaromycesone B (**35**) and the new oxaphenalenone dimer talaromycesone C (**36**) were isolated from the EtOAc extracts of the mycelia of a soil fungus *T. macrosporus* KKU-1NK8. Compound **36** was reported as the second compound of oxaphenalenone dimer with a 5-membered lactone ring on one side (Chaiyosang et al., 2019). Recently, compound **35** was re-isolated from *Talaromyces* sp. IQ-313, an anthill soil-dwelling fungus around the Huasteca Hidalguense, Hidalgo State, Mexico. The cultivation of *Talaromyces* sp. IQ-313 was carried out in potato dextrose agar (PDA) and PDB (Jimenez-Arreolaa et al., 2020).

The cost of production and yield of duclauxin (**1**) on different culture media are different. The production cost of duclauxin (**1**) from *P. herquei* culture on Gostar media (peanut hulls and potato starch substrates) was less than enriched media. However, the yield of duclauxin (**1**) from Gostar medium (7.71 mg/flask) was less than enriched media (8.57 mg/flask). The cost comparison greatly favors the Gostar-type medium for this process (Bryant et al., 1993). Fourteen *in vitro* growth media were used to cultivate the fungi to produce duclauxin analogs in the present report, showing that fungi have different compounds on different artificial growth media under different conditions (Table 1).

## Biological Activities of Duclauxin Metabolites

**FIGURE 7:**
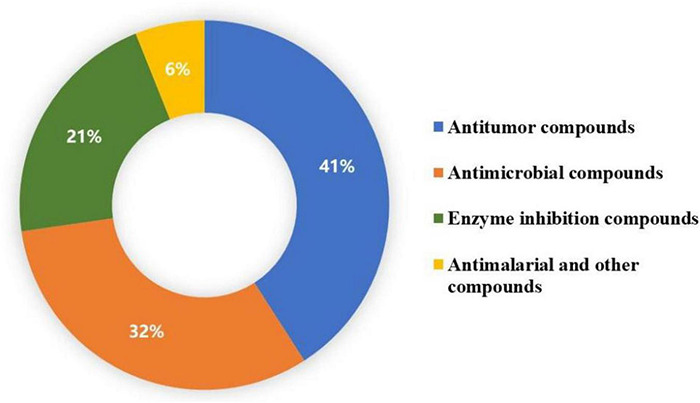
Distribution of biological activities of duclauxin derivatives.

Chemical investigation of genera *Talaromyces* and *Penicillium* led to the isolation of 36 duclauxin analogs, in which only 22 compounds were evaluated for bioactivities. These 22 compounds showed multiple biological activities such as antimicrobial, antimalarial, enzyme inhibition, and antitumor activities. The distribution of bioactive compounds is described in Figure 7. In this section, the detailed bioactivities of duclauxin analogs were summarized, mainly focused on their activities against cancer, infectious disease, Alzheimer’s disease, diabetes, and malaria (Tables 3–6).

**TABLE 3:** Antitumor activities.

Compound	Anti-tumor activities	Activity level	Positive control	References
Duclauxin (**1**)	EAC, L-5178 and HeLa cells	20, 20, and 50 μg/mL (ED_50_)	–	Fuska et al., 1974
	Murine leukemia L1210	3.5 μg/mL (ID_50_)	–	Shiojiri et al., 1983
	Crown gall tumor	–95% at 25 μg/disc-	Camptothecin –100% at 25 μg/disc	Bryant et al., 1994
	MCF-7, NCI-H460 and SF-268	15.0 ± 1.3, 40.7 ± 1.7, and78.3 ± 1.9 μM (GI_50_)	Doxorubin 42.8 ± 8.2, 93 ± 7.0, and 94.0 ± 8.7 μM (GI_50_)	Dethoup et al., 2006
	NS-1 (ATCC TIB-18) mouse myeloma cells	140 μM (IC_50_)	5-Fluorouracil 4.6 μM (IC_50_)	Chaudhary et al., 2020
Bacillisporin A (**2**)	*Vero* cells	34.86 μM (IC_50_)	Ellipticine 3.45 μM (IC_50_)	Chaiyosang et al., 2019
	MCF-7, NCI-H460 and SF-268	10.2 ± 0.9, 7.9 ± 0.3 and 14.7 ± 0.3 μM (GI_50_)	Doxorubin 42.8 ± 8.2, 93 ± 7.0 and 94.0 ± 8.7 μM (GI_50_)	Dethoup et al., 2006
	MCF-7	35.56 μM (IC_50_)	Doxorubicin 23.40 μM (IC_50_) Tamoxifen 27.64 μM (IC_50_)	Dramae et al., 2020
	NCI-H187 and *Vero*	68.06 and 24.81 μM (IC_50_)	Ellipticine 9.87 and 4.34 μM (IC_50_)	
Bacillisporin B (**3**)	*Vero* cells	37.67 μM (IC_50_)	Ellipticine 3.45 μM (IC_50_)	Chaiyosang et al., 2019
	MCF-7	103.12 μM (IC_50_)	Doxorubicin 23.40 μM (IC_50_) Tamoxifen 27.64 μM (IC_50_)	Dramae et al., 2020
	NCI-H187 and *Vero*	90.02 and 78.06 μM (IC_50_)	Ellipticine 9.87 and 4.34 μM (IC_50_)	
	MCF-7, NCI-H460 and SF-286	15.3 ± 1.8, 14.3 ± 1.2, and 21.6 ± 2.1 μM (GI_50_)	Doxorubin 42.8 ± 8.2, 93 ± 7.0, and 94.0 ± 8.7 μM (GI_50_)	Dethoup et al., 2006
Bacillisporin E (**7**)	MCF-7, NCI-H460 and SF-286	81.5 ± 0.6, 85.5 ± 3.4, and > 94.0 μM (GI_50_)	Doxorubin 42.8 ± 8.2, 93 ± 7.0, and 94.0 ± 8.7 μM (GI_50_)	Dethoup et al., 2006
9a-*epi*-Bacillisporin E (**8**)	HeLa cell line	> 100 μM (IC_50_)	Cisplatin 10.6 ± 6.6 μM (IC_50_)	Zang et al., 2016b
Bacillisporin F (**9**)	KB	33.55 μM (IC_50_)	Ellipticine 13.80 μM (IC_50_) Doxorubicin 1.58 μM (IC_50_)	Chaiyosang et al., 2019
	HeLa cell line	> 50 μM (IC_50_)	Cisplatin 10.6 ± 6.6 μM (IC_50_)	Zang et al., 2016b
Macrosporusone D (**14**)	*Vero* cells	37.47 μM (IC_50_)	Ellipticine 3.45 μM (IC_50_)	Chaiyosang et al., 2019
Xenoclauxin (**15**)	L-1210	20 μM	–	Kawai et al., 1985
	*Vero* cells	93.27 μM (IC_50_)	Ellipticine 3.45 μM (IC_50_)	Chaiyosang et al., 2019
Talaromycesone A (**16**)	NIH 3T3 and Hep G2	> 50 μM (IC_50_)	NT	Wu et al., 2015
Talaroketal A (**18**)	Hela cell line	36 ± 2.0 μM (IC_50_)	Cisplatine 5.6 ± 2.0 μM (IC_50_)	Zang et al., 2016a
Talaroketal B (**19**)	Hela cell line	44 ± 2.0 μM (IC_50_)	Cisplatine 5.6 ± 2.0 μM (IC_50_)	Zang et al., 2016a
Bacillisporin H (**21**)	HeLa cell line	49.5 ± 10.3 μM (IC_50_)	Cisplatin 10.6 ± 6.6 μM (IC_50_)	Zang et al., 2016b
Duclauxamide A1(**22**)	HL-60, SMML-7721, A-549, MCF-7, and SW480	11–32 μM (IC_50_)	–	Cao et al., 2015
Duclauxamide B (**23**)	NCI-H187	35.58 μM (IC_50_)	Ellipticine 9.87 μM (IC_50_) Doxorubicin 0.16 μM (IC_50_)	Dramae et al., 2020
Bacillisporin C (**30**)	MCF-7, NCI-H460 and SF-286	26.0 ± 1.0, 37.0 ± 2.5, and 48.0 ± 0.6 μM (GI_50_)	Doxorubin 42.8 ± 8.2, 93 ± 7.0, and 94.0 ± 8.7 μM (GI_50_)	Dethoup et al., 2006
Bacillisporin G (**31**)	NCI-H187, MCF-7 and KB cells	7.29, 9.16, and 5.86 μM (IC_50_)	Doxorubicin 0.35, 17.44, and 1.58 μM (IC_50_)	Chaiyosang et al., 2019
	KB, NCI-H187 and *Vero* cells	5.86, 7.29, and 7.50 μM (IC_50_)	Ellipticine 13.80, 9.26 and 3.45 μM (IC_50_)	
	HeLa cell line	> 50 μM (IC_50_)	Cisplatin 10.6 ± 6.6 μM (IC_50_)	Zang et al., 2016b
Macrosporusone A (**32**)	*Vero* cells	69.77 μM (IC_50_)	Ellipticine 3.45 μM (IC_50_)	Chaiyosang et al., 2019
Macrosporusone B (**33**)	NCI-H187	16.73 μM (IC_50_)	Doxorubicin 0.35 μM (IC_50_) Ellipticine 9.26 μM (IC_50_)	Chaiyosang et al., 2019
	*Vero* cells	13.74 μM (IC_50_)	Ellipticine 3.45 μM (IC_50_)	
Talaromycesone B (**35**)	NIH 3T3 and Hep G2	> 50 μM (IC_50_)	NT	Wu et al., 2015

*“–” means nil.*

*“NT” means not test.*

**TABLE 4:** Antimicrobial activities.

Compound	Antimicrobial activities	Activity level	Positive control	References
Duclauxin (**1**)	*B. subtilis* (ATCC 6633) and *E. coli* (ATCC 25922)	> 200 μM (IC_50_)	Ampicillin 0.9 μM (IC_50_)	Chaudhary et al., 2020
	*Candida albicans* (ATCC 10231) and *Saccharomyces cerevisiae* (ATCC 9763)	> 200 μM (IC_50_)	Ampicillin 0.4 μM (IC_50_)	Chaudhary et al., 2020
Bacillisporin A (**2**)	*S. aureus, S. hemolyticus* and *E. faecalis*	5.2 ± 0.9, 9.5 ± 0.4 and 2.4 ± 0.1 μg/mL (MIC)	Tetracycline 0.05 ± 0.005, 29.5 ± 0.3 and 0.4 ± 0.1 μg/mL (MIC)	Zang et al., 2016b
	*B. subtilis* and *Salmonella*	0.13 ± 0.02 and 2.00 ± 0.02 μM (MIC)	–	Huang et al., 2017
	*B. cereus* and *S. aureus*	3.13 μg/mL (MIC)	Vancomycin 2.0 μg/mL (MIC)	Dramae et al., 2020
	*B. cereus, S. aureus* and MRSA	1.94, 7.75, and 3.88 μM (MIC)	Vancomycin 1.38, 0.35, and 0.35 μM (MIC)	Chaiyosang et al., 2019
	*B. cereus* and *S. aureus*	1.94 and 7.75 μM (MIC)	Kanamycin 4.13 and 2.06 μM (MIC)	
Bacillisporin B (**3**)	*B. cereus* and *S. aureus*	6.25 and 12.50 μg/mL (MIC)	Vancomycin 2.0 and 1.0 μg/mL (MIC)	Dramae et al., 2020
	*B. subtilis*	0.13 ± 0.02 μM (MIC)	–	Huang et al., 2017
Bacillisporin E (**7**)	*B. cereus, S. aureus* and MRSA	3.76, 15.03, and 7.52 μM (MIC)	Vancomycin 1.38, 0.35, and 0.35 μM (MIC)	Chaiyosang et al., 2019
	*B. cereus* and *S. aureus*	3.76 and 15.03 μM (MIC)	Kanamycin 4.13 and 2.06 μM (MIC)	
9a-*epi*-Bacillisporin E (**8**)	*S. aureus, S. hemolyticus* and *E. faecalis*	29.3 ± 0.3, > 30 and > 30 μg/mL (MIC)	Tetracycline 0.05 ± 0.005, 29.5 ± 0.3, and 0.4 ± 0.1 μg/mL (MIC)	Zang et al., 2016b
Bacillisporin F (**9**)	*S. aureus, S. hemolyticus* and *E. faecalis*	15.6 ± 0.5, > 30 and > 30 μg/mL (MIC)	Tetracycline 0.05 ± 0.005, 29.5 ± 0.3, and 0.4 ± 0.1 μg/mL (MIC)	Zang et al., 2016b
Talaromycesone A (**16**)	*S. epidermidis* and *S. aureus* (MRSA)	3.7 and 5.4 μM (IC_50_)	Chloramphenicol 1.81 and 2.46 μM (IC_50_)	Wu et al., 2015
Talaroketal A (**18**)	*S. aureus*	50 μg/mL (IC_50_)	Tetracycline 0.077 ± 0.5 μg/mL (IC_50_)	Zang et al., 2016a
Talaroketal B (**19**)	*S. aureus*	50 μg/mL (IC_50_)	Tetracycline 0.077 ± 0.5 μg/mL (IC_50_)	Zang et al., 2016a
Bacillisporin H (**21**)	*S. aureus*, and *S. hemolyticus* and *E. faecalis*	5.0 ± 0.9, 20.4 ± 6.5, and > 30 μg/mL (MIC)	Tetracycline 0.05 ± 0.005, 29.5 ± 0.3, and 0.4 ± 0.1 μg/mL (MIC)	Zang et al., 2016b
Duclauximide B (**23**)	*M. tuberculosis*	12.5 μg/mL (MIC)	Streptomycin 0.62 μg/mL (MIC)	Dramae et al., 2020
	*S. aureus* and *B. cereus*	12.5 mg/mL (MIC)	Vancomycin 2.0 and 1.0 μg/mL (MIC)	Dramae et al., 2020
Bacillisporin C (**30**)	*B. cereus* and *S. aureus*	25.0 and 50.0 μg/mL (MIC)	Vancomycin 2.0 and 1.0 μg/mL (MIC)	Dramae et al., 2020
Bacillisporin G (**31**)	*S. aureus*, and *S. hemolyticus* and *E. faecalis*	> 50 μg/mL (MIC)	Tetracycline 0.05 ± 0.005, 29.5 ± 0.3, and 0.4 ± 0.1 μg/mL (MIC)	Zang et al., 2016b
Talaromycesone B (**35**)	*S. epidermidis* and *S. aureus* (MRSA)	17.36 and 19.50 μM (IC_50_)	Chloramphenicol 1.81 and 2.46 μM (IC_50_)	Wu et al., 2015

*“–” means nil.*

**TABLE 5:** Enzyme inhibition activities.

Compound	Enzyme inhibition activities	Activity level	Positive control	References
Duclauxin (**1**)	*h*PTP1B1-400	12.7 μM (IC_50_)	Ursolic acid 26.7 μM (IC_50_)	Jimenez-Arreolaa et al., 2020
	Anti-EGFR	0.95 ± 0.64 μM (IC_50_)	Afatinib 0.0005 ± 0.00002 μM (IC_50_)	Wang et al., 2019
	Anti-CDC25B	0.75 ± 0.18 μM (IC_50_)	Na_3_VO_4_ 0.52 ± 0.02 μM (IC_50_)	Wang et al., 2019
Bacillisporin A (**2**)	α-Glucosidase	33.55 ± 0.63 μM (IC_50_)	Acarbose 1075.53 ± 11.94 μM (IC_50_)	Huang et al., 2017
Bacillisporin B (**3**)	α-Glucosidase	95.81 ± 1.12 μM (IC_50_)	Acarbose 1075.53 ± 11.94 μM (IC_50_)	Huang et al., 2017
Bacillisporin F (**9**)	Anti-CDC25B	0.40 ± 0.02 μM (IC_50_)	Na_3_VO_4_ 0.52 ± 0.02 μM (IC_50_)	Wang et al., 2019
	Anti-EGFR	4.41 ± 2.32 μM (IC_50_)	Afatinib 0.0005 ± 0.00002 μM (IC_50_)	Wang et al., 2019
Xenoclauxin (**15**)	Anti-CDC25B	0.26 ± 0.06 μM (IC_50_)	Na_3_VO_4_ 0.52 ± 0.02 μM (IC_50_)	Wang et al., 2019
	Anti-EGFR	0.24 ± 0.17 μM (IC_50_)	Afatinib 0.0005 ± 0.00002 μM (IC_50_)	Wang et al., 2019
	*h*PTP1B_1–400_	21.8 μM (IC_50_)	Ursolic acid 26.7 μM (IC_50_)	Jimenez-Arreolaa et al., 2020
Talaromycesone A (**16**)	*AChE*	7.49 μM (IC_50_)	Huperzine 11.6 μM (IC_50_)	Wu et al., 2015
Verruculosin B (**17**)	Anti-EGFR	1.22 ± 0.53 μM (IC_50_)	Afatinib 0.0005 ± 0.00002 μM (IC_50_)	Wang et al., 2019
Verruculosin A (**20**)	Anti-CDC25B	0.38 ± 0.03 μM (IC_50_)	Na_3_VO_4_ 0.52 ± 0.02 μM (IC_50_)	Wang et al., 2019
	Anti-EGFR	0.92 ± 0.25 μM (IC_50_)	Afatinib 0.0005 ± 0.00002 μM (IC_50_)	Wang et al., 2019
Bacillisporin G (**31**)	*h*PTP1B_1–400_	13.4 μM (IC_50_)	Ursolic acid 26.7 μM (IC_50_)	Jimenez-Arreolaa et al., 2020
Talaromycesone B (**35**)	*h*PTP1B_1–400_	82.1 μM (IC_50_)	Ursolic acid 26.7 μM (IC_50_)	Jimenez-Arreolaa et al., 2020

*“NT” means not test.*

*“Na_3_VO_4_” stands for trisodium vanadate.*

**TABLE 6:** Antimalarial and others activities.

Compound	Antimalarial and others activities	Activity level	Positive control	References
Duclauxin (**1**)	Stimulatory biological activity against etiolated wheat coleoptiles	100% at 10^–3^ M	–	Bryant et al., 1993
Bacillisporin G (**31**)	Antimalarial activity against *P. falciparum*	8.07 μM (IC_50_)	Dihydroartemisinin 0.003 μM (IC_50_)	Chaiyosang et al., 2019
Macrosporusone B (**33**)	Antimalarial activity against *P. falciparum*	10.28 μM (IC_50_)	Dihydroartemisinin 0.003 μM (IC_50_)	Chaiyosang et al., 2019

*“–” means nil.*

### Antitumor Activities

According to the World Health Organization (WHO), cancer is a hazardous disease primarily caused by malignant tumors resulting from abnormal cell growth. Cancer is a leading cause of death around the globe; nearly 10 million people have died from cancer in 2020 worldwide (World Health Organization, 2021).^1^ Various techniques have been used to control cancer, such as radiotherapy, chemotherapy, and surgery. Among these techniques, chemotherapy is the most helpful approach to treat cancer so far (Farce et al., 2004). Therefore, finding effective antitumor agents from a natural source is necessary due to their lower toxicity.

Duclauxin (**1**), a metabolite that occurs naturally in fungi, is the most effective against numerous tumor cell lines because it prevents ATP synthesis by inhibiting mitochondrial respiration and therefore exhibits potential as a leading anticancer molecule. The antitumor activity of duclauxin (**1**) was evaluated *in vitro* against Ehrlich ascites carcinoma (EAC), lymphadenoma L-5178, and HeLa cells. The inhibitory effect was evaluated according to the decreased number of nucleic acids in tested tumor cells. It suppressed the growth of all tested tumor cell lines with cytotoxic effect ED_50_ from 20 to 50 μg/mL (Fuska et al., 1974). Duclauxin (**1**) was evaluated for the effect on murine leukemia L1210 culture cells, and it was strongly active with the ID_50_ value of ca. 3.5 μg/mL (6.6 μM) (Shiojiri et al., 1983). Similarly, duclauxin (**1**) was examined using an *Agrobacterium tumefaciens* potato disc assay for crown gall tumor/antitumor induction at the concentrations of 5 μg/disc, 10 μg/disc, 25 μg/disc, and 50 μg/disc, respectively. The inhibitory potential values at the given concentrations were –67, –74, –95, and –94%, respectively. This observation concurs that duclauxin (**1**) was an effective antitumor agent at the concentrations of 25 μg/disc (camptothecin, positive control, –100% at 25 μg/disc) (Bryant et al., 1994). Duclauxin (**1**) isolated from *T*. *bacillisporus* was intensely active against three human cancer cell lines (MCF-7, NCI-H460 and SF-286) with the following GI_50_ values of 15.0 ± 1.3, 40.3 ± 1.7, and 78.3 ± 1.9 μM, respectively. However, all the cancer cell lines were less inhibited by the positive control doxorubin with the GI_50_ values of 42.8 ± 8.2, 93 ± 7.0, and 94.0 ± 8.7 μM, respectively (Dethoup et al., 2006). The cytotoxicity of duclauxin (**1**) was recently examined against NS-1 (ATCC TIB-18) mouse myeloma cells and found to be inactive (Chaudhary et al., 2020; Table 3).

Duclauxamide A1 (**22**) isolated from *P. manginii*, exhibited moderate cytotoxicity against SW480, MCF-7, A-549, MCF-7 SMML-7721, and HL-60 cancer cell lines with inhibitory concentration (IC_50_) values in the range of 11–32 μM (Cao et al., 2015). The cytotoxic properties of compounds **8, 9, 21,** and **31** were determined against HeLa cell line by the MTT assay, in which cisplatin was used as a positive control. Only bacillisporin H (**21**) displayed modest cytotoxicity against the HeLa cell line with an IC_50_ value of 49.5 μM, while the other compounds were inactive (cisplatin, positive control, IC_50_: 10.6 ± 6.6 μM) (Zang et al., 2016b). Biological evaluation against HeLa cell line showed that talaroketals A and B (**18–19**) exhibited moderate cytotoxicity with IC_50_ values 36 ± 2.0 μM and 44 ± 2.0 μM, respectively (cisplatine, positive control, IC_50_: 5.6 ± 2.0 μM) (Zang et al., 2016a). Kawai et al. (1985) assessed the *in vitro* cytotoxicity effect of xenoclauxin (**15**) against murine leukemia (L-1210 culture cells), and it exhibited the strongest inhibitory activity by causing a complete growth inhibition at approximately 20 μM. Compound **15** exhibited the same mode of inhibition as that of duclauxin on the ATP synthesis in mitochondria, displaying uncoupling of oxidative phosphorylation and inhibition of state 3 respiration (Kawai et al., 1985; Table 3).

Oxaphenalenones **2–3**, **7**, and **30** were screened for *in vitro* cytotoxicity against three human cancer cell lines MCF-7, NCI-H460, and SF-286. Among them, bacillisporin A (**2**) showed strong growth inhibitory effects against MCF-7 and NCI-H460 with GI_50_ values of 10.2 ± 0.9 and 7.9 ± 0.3 μM, respectively. It also exhibited a moderate GI_50_ on SF-268 with a value of 14.7 ± 0.3 μM. Bacillisporins B (**3**) and C (**30**) also showed an average mild growth inhibitory effect against all cancer cell lines (MCF-7, NCI-H460, and SF-286) with GI_50_ from 15.0 ± 1.3 to 78.3 ± 1.9 μM. However, bacilisporin E (**7**) exhibited a weak activity compared to the positive control and other tested compounds. GI_50_ values of these compounds were determined by the National Cancer Institute *in vitro* anticancer molecule discovery screen using protein-binding dye sulforhodamine B (SRB) (Skehan et al., 1990; Dethoup et al., 2006). In another study, bacillisporins A-B (**2**–**3**) and duclauxamide B (**23**) were evaluated against cancerous (MCF-7 and NCI-H187) and non-cancerous (*Vero*) cells. Only compound **2** displayed notable activity against the MCF-7 cell line with an IC_50_ value of 35.56 μM as compared to positive control tamoxifen (IC_50_, 27.64 μM), while compound **3** demonstrated weak cytotoxicity against both cancerous and non-cancerous cells with IC_50_ values ranging from 78.06 to 103.12 μM. In contrast, duclauxamide B (**23**) demonstrated moderate cytotoxicity exclusively against NCI-H187 with IC_50_ 35.58 μM (ellipticine, positive control, IC_50_: 9.87 μM, Table 3; Dramae et al., 2020).

Macrosporusone B (**33**) exhibited significant cytotoxic activity against NCI-H187 (human small cell lung cancer) and primate (*Vero*) cells with the IC_50_ values of 16.73 and 13.74 μM, respectively (ellipticine, positive control, IC_50_: 9.26 and 3.45 μM). At the same time, bacillisporin G (**31**) showed remarkable cytotoxic activity against NCI-H187, MCF-7 (human breast adenocarcinoma), KB (human epidermoid carcinoma in the mouth) and primate (*Vero*) cell lines with IC_50_ values of 7.29, 9.16, 5.86, and 7.50 μM, respectively. In this study, ellipticine was used as a positive control, displayed cytotoxicity againstNCI-H187, KB, and *Vero* cells with the IC_50_ values of 9.26, 13.80, and 3.45 μM, respectively. The IC_50_ value of standard control doxorubicin against the MCF-7 cell line was 17.44 μM. This information concurs that compound **31** might be used as a leading hit against the cancer lines. Furthermore, compounds **2**, **3**, **14–15**, and **32** exhibited weak cytotoxicity against *Vero cells* with IC_50_ values in the range of 33.55–93.27 μM. In comparison, the KB cell line was slightly inhibited by compound **9** with the IC_50_ of 33.55 μM (ellipticine, positive control, IC_50_: 13.80 μM). Resazurin microplate assay (REMA) was employed to perform the activity against NCI-H187, MCF-7, and KB cell lines. In addition, cytotoxicity against *Vero* cells was tested using the green fluorescent protein (GFP) detection method (Chaiyosang et al., 2019). Talaromycesones A (**16**) and B (**35**) isolated from marine fungus *Talaromyces* sp. LF458 were evaluated for cytotoxic activity against HepG2 (human hepatocellular liver carcinoma cell line) and NIH 3T3 (mouse fibroblasts cell line). Both compounds were inactive against tested cancer cell lines (Wu et al., 2015; Table 3).

### Antimicrobial Activities

Antimicrobial resistance (AMR) has emerged as one of the most serious human health concerns of the twenty-first century. The Center for Disease Control and Prevention (CDC) reported that 23,000 people die every year in developing countries due to AMR. The emergence of multidrug-resistant strains, such as methicillin-resistant *Staphylococcus aureus* (MRSA), significantly contributes to AMR incidence. Antimicrobials are the most effective way to protect human beings from infectious diseases (Basak et al., 2016; Cruz et al., 2020; Health Research and Educational Trust, 2020).^2^ Thus, fungi-derived secondary metabolites are a potential pool for antimicrobial agent screening, which holds a great promise for new antibiotics.

Duclauxin (**1**) was evaluated against pathogenic bacteria and fungi using the 96-well microtiter plate method. However, it was not active against *Bacillus subtilis* ATCC 6633, *Escherichia coli* ATCC 25922, *Candida albicans* ATCC 10231, and *Saccharomyces cerevisiae* ATCC 9763, respectively (Chaudhary et al., 2020). Bacillisporins A (**2**) and B (**3**) were inactive against *Acinetobacter baumannii*, *Escherichia coli*, *Pseudomonas aeruginosa*, and *Enterococcus faecium* at a concentration of 50 μg/mL, but bacillisporin A (**2**) possessed potent antibacterial activity against Gram-positive bacteria such as *S. aureus* and *B. cereus* with the same MIC value of 3.13 μg/mL (vencomycin, positive control, MIC: 2.0 and 1.0 μg/mL). However, bacillisporin B (**3**) exhibited moderate antibacterial activity against *B. cereus* and *S. aureus* with MIC values of 6.25 and 12.50 μg/mL, respectively (vencomycin, positive control, MIC: 2.0 and 1.0 μg/mL), while bacillisporin C (**30**) displayed weak antibacterial activity against both strains with MIC values of 25.0 and 50.0 μg/mL, respectively. Duclauximide B (**23**), a *N*-containing oxaphenalenone dimer, displayed moderate inhibitory activity against *Mycobacterium tuberculosis, S. aureus* and *B. cereus* with the same MIC value of 12.5 μg/mL. The standard positive control values against *Mycobacterium tuberculosis, S. aureus* and *B. cereus* are listed in Table 4 (Dramae et al., 2020). In another report, bacillisporins A (**2**) and B (**3**) were tested for antimicrobial activities against *B. cereus*, *Sarcina lutea*, *S. albus*, *E. coli*, and *Salmonella*, using the microplate assay method. Both compounds displayed strong inhibitory activities against *B. subtilis* with the same MIC values of 0.13 ± 0.02 μM, whereas the MIC value of compound **2** against *Salmonella* was 2.00 ± 0.02 μM (Huang et al., 2017). Substantial antibacterial activities against clinically relevant pathogenic bacterial strains *S. epidermidis* DSM 20044 and methicillin-resistant *S. aureus* (MRSA) DSM 18827 were observed for talaromycesone A (**16**) with an IC_50_ 3.70 and 5.4 μM, respectively (chloramphenicol, positive control, IC_50_, 1.81 and 2.46 μM). At the same time, talaromycesone B (**35**) showed moderate inhibitory effects against *S. epidermidis* and *S. aureus* with IC_50_ values of 17.36 and 19.50 μM, respectively (Wu et al., 2015; Table 4).

Compounds **2, 3, 7, 9, 15**, **31–33**, and **35** isolated from *T. macrosporus* KKU-1NK8, evaluated for antibacterial activity against Gram-positive (*B. cereus* ATCC 11778, *S. aureus* ATCC 6538, MRSA) and Gram-negative bacteria (*E. coli* ATCC 25922, *P. aeruginosa* ATCC 27853, and *Salmonella enterica* serovar Typhimurium ATCC 13311). Among them, bacillisporins A (**2**) and E (**7**) displayed the most vigorous antibacterial activities against *B. cereus* with MIC of 1.94 and 3.76 μM, respectively. Therefore, they can be used as anti-*B. cereus* candidate molecule due to their intense effects compared to standard drugs vancomycin (MIC 1.38 μM) and kanamycin (MIC 4.13 μM), respectively. Moreover, compound (**3**) was slightly active against Gram-positive bacteria, while the other tested compounds were inactive against Gram-positive and Gram-negative bacteria. The dilution method was used to evaluate the minimum inhibitory concentrations (MICs), as described in the M07-A9 of the Clinical and Laboratory Standards Institute (Balouiri et al., 2016; Chaiyosang et al., 2019; Table 4).

Talaroketals A (**18**) and B (**19**) were examined for their antimicrobial activities against four strains such as *E. coli* ATCC 8739, *S. aureus* ATCC 6538, *S. haemolyticus* MNHN26, and *Enterococcus faecalis* CIP 103014. These compounds exhibited weak antimicrobial activity against *S. aureus* with an IC_50_ value of around 50 μg mL^–1^ but no action against the other bacterial strains (Zang et al., 2016a). Bacillisporins A (**2**), F (**9**), G (**31**), and H (**21**) were evaluated against human pathogenic bacteria *E. coli* ATCC 8739, *S. aureus* ATCC 6538, *S. hemolyticus* MNHN26, and *E. faecalis* CIP 103014. Among them, bacillisporins A (**2**) and H (**21**) revealed potent antimicrobial activity against *S. hemolyticus* with MIC values of 9.5 ± 0.4 and 20.4 ± 6.5 μg/mL, respectively. Tetracycline, the positive control (MIC 29.5 ± 0.3 μM), was less active than the compounds **2** and **21**. The MIC value of bacillisporin H (**21**) against *S. aureus* was 5.0 μg/mL, and the other compounds displayed weak activity against the panel of human pathogenic bacteria (Zang et al., 2016b; Table 4).

### Enzyme Inhibition Activities

**FIGURE 8:**
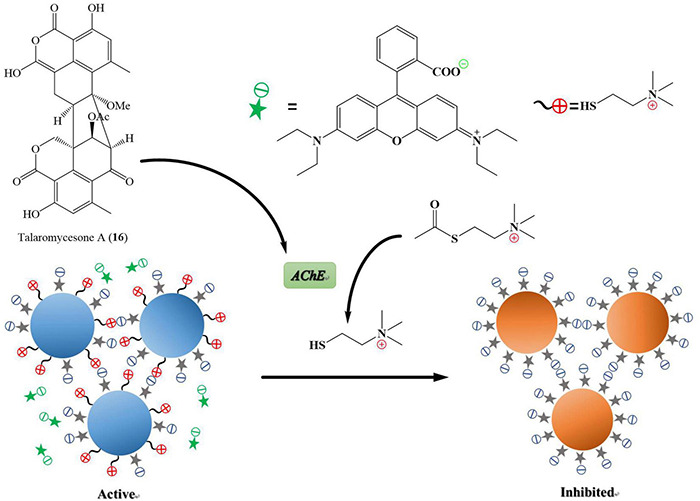
Acetylcholinesterase (*AChE*) is an enzyme that catalyzes acetylcholine into choline and acetate, resulting in the termination of synaptic transmission, responsible for Alzheimer’s disease. *AChE* is an enzymatic target for the treatment of Alzheimer’s disease. It has been shown in Figure 7 that the compound named talaromycesone A (**16**) inhibited the enzyme *AChE* from breaking down acetylcholine into choline and acetate.

Alzheimer’s disease is a progressive neurodegenerative disease that causes brain cells to waste away and die. It is characterized by progressive cognitive deterioration and continuous decline in thinking, behavioral, and social skills that disrupts a person’s ability to function independently (Khan et al., 2016). Around 50 million people are affected by Alzheimer’s disease across the globe, and it is likely to present an increasing demand for the innovation of small molecules for this type of dementia (World Health Organization, 2020).^3^ Acetylcholinesterase (*AChE*) is an enzyme that catalyzes acetylcholine hydrolysis. Inhibition of this enzyme leads to an increased level of the neurotransmitter acetylcholine, which improves cholinergic functions in patients (Li et al., 2019). Talaromycesone A (**16**), isolated from the broth and mycelia of a marine fungus *Talaromyces* sp. LF458 was the first oxaphenalenone structure that exhibited *AChE* inhibitory activity with an IC_50_ of 7.49 μM (huperzine, positive control, IC_50_ 11.6 μM). Therefore, compound **16** is a potential *AChE* inhibitor that could be a candidate molecule against Alzheimer’s disease (Figure 8; Wu et al., 2015).

Bacillisporins A (**2**) and B (**3**) produced by *T. aculeatus* exhibited potent inhibitory activity against α-glucosidase enzyme with IC_50_ values of 33.55 ± 0.63 and 95.81 ± 1.12 μM, respectively (acarbose, positive control, IC_50_ 1075.53 ± 11.94 μM). The α-glucosidase inhibition activity of these compounds was determined using the method described by Jeon et al. (2013) with slight modifications (Huang et al., 2017; Table 4). Novel molecules against α-glucosidase are urgently needed because of their essential role in glucose absorption in the gastrointestinal tract, the inhibition of which prevents postprandial hyperglycemia. Compounds **2** and **3** are potential candidates of lead compounds for the treatment of type II diabetes.

Oligophenalenone dimers such as **1**, **9**, **15**, **17,** and **20** were assayed for their effects against cell division cycle 25B (CDC25B) tyrosine phosphatase. Among them, verruculosin A (**20**), bacillisporin F (**9**), and xenocluaxin (**15**) showed strong inhibitory activity against CDC25B with IC_50_ values of 0.38 ± 0.03, 0.40 ± 0.02, and 0.26 ± 0.06 μM, respectively (Na_3_VO_4_, positive control, IC_50_ 0.52 ± 0.02 μM). The CDC25B regulates cell cycle progression in various cancer cell lines and has contributed to tumor growth. However, the specific mechanism by which increased CDC25B impacts tumor progression is not clear. The results indicated that oligophenalenone dimers might be used for screening as the new CDC25B inhibitor candidates. In addition, compounds **1**, **9**, **15**, **17,** and **20** displayed weak inhibitory activity toward epidermal growth factor receptor (EGFR) tyrosine kinase with IC_50_ values ranging from 0.92 ± 0.25 to 4.41 ± 2.32 μM (afatinib, positive control, IC_50_ 0.0005 ± 0.00002 μM) (Wang et al., 2019; Table 5).

A critical biological event that contributes to the appearance and progress of cancer and diabetes is the reversible phosphorylation of proteins, a process controlled by protein tyrosine phosphatase (PTPs). Within the PTPs, PTP1B has gained significant interest from a pharmacological point of view since it is an emerging therapeutic target in treating cancer and diabetes. Until now, more than 300 PTP1B_1–300_ inhibitors have been identified from nature, including microorganisms and plants (Verma et al., 2017). Particularly, chemical investigation of a soil fungus *Talaromyces* sp. IQ-313 has resulted in the isolation of many different types of PTP1B inhibitors, such as duclauxin (**1**), bacillisporin G (**31**), and xenoclauxin (**15**), which showed potent inhibitory activity toward human protein tyrosine phosphatase 1B (*h*PTP1B_1–400_) in a concentration-dependent manner with IC_50_ values of 12.7, 13.4, and 21.8 μM, respectively (Ursolic acid, positive control, IC_50_ 26.7 μM). These compounds induce conformational changes in the protein, which directly affect its activity, suggesting these compounds are allosteric modulators of the protein. Thus, compounds **1**, **15**, and **31** have great potential to treat obesity and chronic diseases such as diabetes and cancer (Jimenez-Arreolaa et al., 2020; Table 5).

### Antimalarial and Other Activities

Malaria is a parasitic disease caused by *Plasmodium* spp. resulting from the bites of infected mosquitoes. Approximately 220 million people are infected by malaria, resulting in 435,000 deaths worldwide per annum. The emergence of drug-resistant malaria strains to classical synthetic drugs requires a continuous search for new compounds from alternative niches to introduce new and efficient therapies (Cadamuro et al., 2021). Bacillisporin G (**31**) and macrosporusone B (**33**) produced by the fungus *T. macrosporus* displayed weak antimalarial activity against *Plasmodium falciparum* (IC_50_ values were 8.07 and 10.28 μM, respectively) in comparison with positive control dihydroartemisinin (IC_50_ value was 0.003 μM) (Chaiyosang et al., 2019). It was observed that duclauxin (**1**) significantly inhibited the growth of etiolated wheat coleoptiles in a concentration-dependent manner. The percentage of inhibition at selected concentrations of duclauxin relative to controls were: 100% at 10^–3^ M, 80% at 10^–4^ M, 39% at 10^–5^ M, and 0% at 10^–6^ M (Bryant et al., 1993; Table 6).

## Biosynthesis of Duclauxin Derivatives

**FIGURE 9:**
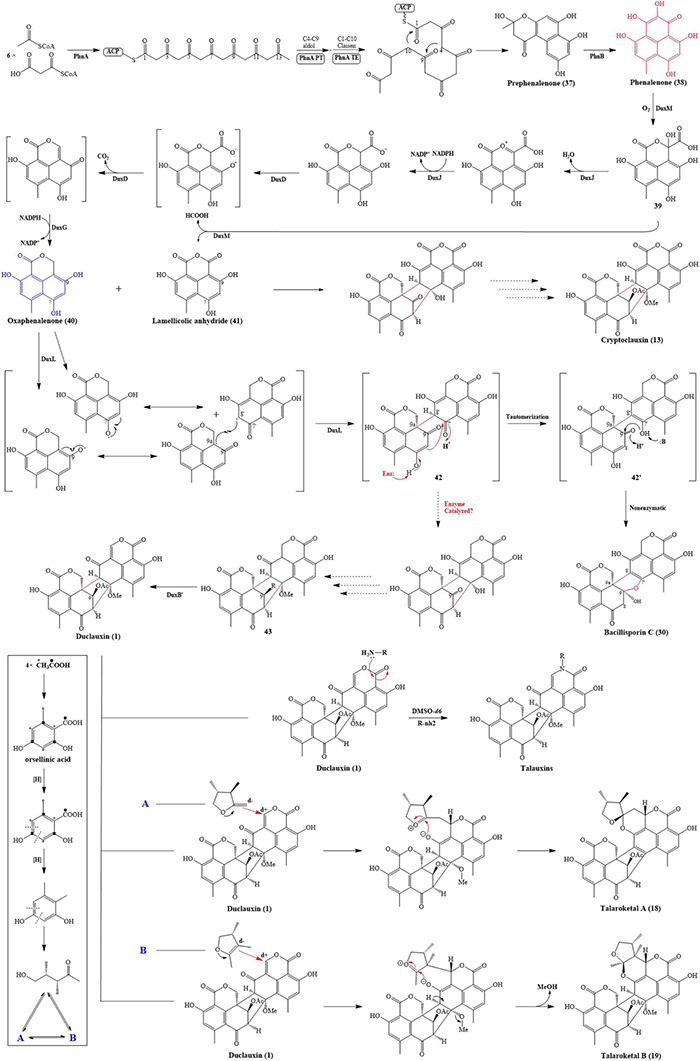
Proposed biosynthetic pathway of duclauxin (**1**) and its derivatives.

The biosynthetic investigations of duclauxin and its derivatives have been partially clarified. Gao and co-workers studied the biosynthesis of duclauxin (**1**) at the molecular level and identified the respective responsible biosynthetic gene clusters in *P. herquei* and *T. stipitatus* (Gao et al., 2016, 2018). Briefly, biosynthesis of duclauxin (**1**) includes three extensive redox steps: (i) The phenalenone (**38**) is biosynthesized through redox reactions from an acetyl-CoA and malonyl-CoAs as the first key intermediate in duclauxin biosynthesis; (ii) A series of oxidordeuctases catalyze redox reactions on phenalenone (**38**) to yield the second key intermediate oxaphenalenone (SF226, **40**); (iii) Dimerization of SF226 and/or lamellicolic anhydride can yield duclauxin (**1**) and its derivatives as shown in Figure 9.

The phenalenone (**38**) is a key intermediate in duclauxin biosynthesis (Figure 9). Phenalenone (**38**) is an aromatic polyketide with a 6/6/6 ring system essential in biosynthetic studies. In *P. herquei*, a gene cluster named *phn* was associated with phenalenone (**38**) production, and only two genes in this cluster, *phnA* and *phnB*, were sufficient for the formation of phenalenone (**38**). *phnA* encoding a non-reducing polyketide synthase, which has a domain architecture of SAT-KS-MAT-PT-ACP-ACP-TE-CLC (Gao et al., 2016), used 6 malonyl-CoAs and an acetyl-CoA as building blocks to generate the prephenalenone (**37**) core. During this process, the PT domain of *phnA* catalyzed the aldol condensation of C4-C9 cyclization of the polyketide chain, and the TE/CLC domain was responsible for C1-C10 Claisen condensation (Gao et al., 2016). After the formation of prephenalenone (**37**), a FAD-dependent hydroxylase encoded by *phnB* was used to form phenalenone (**38**) (Gao et al., 2016). Using phenalenone (**38**) as an essential precursor or intermediate, enzymes encoded by downstream genes facilitate redox reactions on phenalenone to yield diversified duclauxin analogs. In *T. stipitatus*, a gene cluster named *dux1* possessed two counterparts to *phnA* and *phnB*: *duxI* and *duxE*, and they were likewise responsible for phenalenone formation. In this gene cluster, some additional genes were also found, including predicted P450 monooxygenases (DuxD and DuxL), oxidoreductase (DuxB), NAD(P)H-dependent reductases (DuxA, DuxG, and DuxJ), and a cupin family oxygenase (DuxM), transcription factor (DuxC), hydrolase (DuxH), and *O*-methyltransferase (DuxK) (Gao et al., 2018). Firstly, DuxM catalyzed phenalenone to another important intermediate **39**, **39** can be further transformed to lamellicolic anhydride (**41**) by DuxM. Secondly, a series of sequential redox reactions catalyzed by DuxJ, DuxD and DuxG took place on **39** to yield SF226 (**40**), another key building block of duclauxin and its analogs (Gao et al., 2018). After the formation of oxaphenalenone (**40**) and lamellicolic anhydride (**41**), mediated by P450 monooxygenase DuxL through a diradical-coupling mechanism, two oxaphenalenones (**40**) formed dimer **42**, which can finally nonenzymatically convert to bacillisporin C (**30**), or possibly enzymatically convert to **43** (Gao et al., 2018; Liu et al., 2021). However, further functional experiments are required to prove it. Intriguingly, in *T. stipitatus*, two identical genes (*duxB* and *duxB’*) were involved in transforming **43** to duclauxin (**1**). In comparison, a molecule of oxaphenalenone (**40**) and a molecule of lamellicolic anhydride (**41**) can finally lead to the formation of cryptoclauxin (**13**) (Gao et al., 2018).

Zang et al. (2016a) proposed the biosynthetic pathway for talaroketals A and B (**18** and **19**). In short, the upper 6/6/6 tri-ring system of duclauxin (**1**) can be modified into duclauxin derivatives (Figure 9). For example, a nucleophilic species can react with the upper ring to give the spiroketal moiety of talaroketal A (**18**) and the fused ketal part of talaroketal B (**19**) (Zang et al., 2016a). In another case, the upper moiety present in the structure of heptacyclic duclauxin (**1**) can react with different amino acids to yield talauxins Q, L, and I (**26**, **28–29**), which could happen without the presence of specific enzymes (Figure 9; Chaudhary et al., 2020). The amino acids present in these talauxins possess hydrophobic and hydrophilic side chains, indicating that other amino acids probably react with duclauxin (**1**) to produce more talauxin derivatives.

## Conclusion and Prospect

Over the past few years, about 36 duclauxin derivates have been identified from at least 9 fungal species mainly distributed in the genera *Penicillium* and *Talaromyces* (*T. duclauxii*, *T. aculeatus*, *T. stipitatus*, *T. bacillisporus*, *T. verruculosus*, *T. macrosporus*, *P. herquei*, *P. manginii*, and *Talaromyces* sp.). These fungal species were isolated from various niches, including soil, mangroves, plants endophytes, and marine ecosystem. According to the latest nomenclature and phylogenetic analysis, *Penicillium* species classified in the subgenus *Biverticillium* belong to genus *Talaromyces.* The species such as *P. duclauxii*, *P. aculeatum*, and *P. stipitatum* involved in duclauxins isolation are now named *T. duclauxii*, *T. aculeatus* and *T. stipitatus*, respectively. In recent years, significant strides of progress have been made in deciphering the mechanism and functions of duclauxin and its derivatives. Numerous studies showed that duclauxin derivatives revealed various significant biological activities, including antitumor, antimicrobial, and enzyme inhibition. Talaromycesone A (**16**), isolated from marine fungus *Talaromyces* sp. LF458, is the first oxaphenalenone with potent *AChE* inhibitory activity, signifying its potential for further research against Alzheimer’s disease. Similarly, bacillisporin A (**2**) is critically important to the development of leading hit for the treatment of type II diabetes, owing to its inhibitory activity against α-glucosidase, an enzyme responsible for postprandial hyperglycemia. Until now, to the best of our knowledge, total synthesis has not been successfully achieved to construct the delicate architectures of duclauxin derivatives. However, in this review, important information was provided in discovering essential genes and enzymes responsible for the biosynthesis of duclauxin, which would be helpful to achieve novel duclauxin derivatives biosynthetically.

Modern experimental approaches and computing tools are expected to unravel mechanisms of action (MOA) of duclauxins. Techniques such as RNA-seq and bioinformatics would provide a theoretical basis for the MOA study of duclauxins. Research to date indicates that more profound studies are needed to understand the biosynthetic pathway, metabolic regulation, and structure-activity relationship of duclauxins. Lastly, the physiological and ecological roles of the duclauxin metabolites and their practical applications in medicine, forestry, and agriculture will be confidently expected.

## Author Contributions

HS, PD, and TS performed the review design. TC and YW performed the drawing of the compounds structure. HS and YY constructed the phylogenetic tree. HS and TS completed the manuscript draft. YW, CZ, and ZM finished the review and editing. All authors have read and agreed to the published version of the manuscript.

## Conflict of Interest

The authors declare that the research was conducted in the absence of any commercial or financial relationships that could be construed as a potential conflict of interest.

## Publisher’s Note

All claims expressed in this article are solely those of the authors and do not necessarily represent those of their affiliated organizations, or those of the publisher, the editors and the reviewers. Any product that may be evaluated in this article, or claim that may be made by its manufacturer, is not guaranteed or endorsed by the publisher.

## Footnotes

1
https://www.who.int/news-room/fact-sheets/detail/cancer

2
www.hret-hiin.org

3
https://www.who.int/news-room/fact-sheets/detail/dementia
